# Trophoblast Differentiation: Mechanisms and Implications for Pregnancy Complications

**DOI:** 10.3390/nu15163564

**Published:** 2023-08-12

**Authors:** Lauren Lawless, Yushu Qin, Linglin Xie, Ke Zhang

**Affiliations:** 1 Institute of Bioscience and Technology, Texas A&M University, Houston, TX 77030, USA; labedrich@tamu.edu; 2 Department of Nutrition, Texas A&M University, College Station, TX 77843, USA

**Keywords:** placental development, trophoblast differentiation, pregnancy, preeclampsia, maternal health

## Abstract

Placental development is a tightly controlled event, in which cell expansion from the trophectoderm occurs in a spatiotemporal manner. Proper trophoblast differentiation is crucial to the vitality of this gestational organ. Obstructions to its development can lead to pregnancy complications, such as preeclampsia, fetal growth restriction, and preterm birth, posing severe health risks to both the mother and offspring. Currently, the only known treatment strategy for these complications is delivery, making it an important area of research. The aim of this review was to summarize the known information on the development and mechanistic regulation of trophoblast differentiation and highlight the similarities in these processes between the human and mouse placenta. Additionally, the known biomarkers for each cell type were compiled to aid in the analysis of sequencing technologies.

## 1. Introduction

During pregnancy, the placenta plays a vital role in ensuring optimal fetal growth and development by facilitating the exchange of essential nutrients, oxygen, and waste products between the mother and the fetus. This temporary organ is formed when placental cells infiltrate the uterine arteries, establishing contact with the maternal circulation and redirecting blood flow to the placenta. The placenta contains a specialized vascular network that relies on diffusion and transport mechanisms to carry out its functions, maintaining a barrier between maternal and fetal blood.

Proper development of the placenta relies on intricate processes of cell differentiation. As trophoblast cells differentiate into their terminal cell types, critical events, such as spiral artery remodeling and vascularization, take place, enabling the formation of a functional placenta. Spiral artery remodeling involves the transformation of maternal arteries into high-velocity, low-resistance vessels by invasive trophoblast cells, facilitating sufficient blood flow into the placenta. Concurrently, placental vascularization establishes a specialized network of fetal capillaries that interact with the maternal circulation to extract vital nutrients and oxygen necessary for fetal growth.

Disruptions in these developmental processes can lead to various complications, including fetal growth restriction, preeclampsia, preterm birth, delayed physical development, reduced cognitive function in the fetus, and increased disease risk in postnatal life [1,2,3,4,5]. Additionally, mothers may experience cardiovascular disease and kidney dysfunction after giving birth [6,7,8,9,10]. While the underlying causes of these placenta-related complications are not yet fully understood, placental hypoxia resulting from inadequate maternal vasculature remodeling is considered a plausible explanation [11,12]. Currently, the only known treatment for many of these complications is the delivery of the placenta, underscoring the need for further investigation into the processes contributing to the pathophysiology of these diseases [13].

As previously mentioned, trophoblast differentiation governs the initiation and sufficiency of the developmental processes required for a functional placenta. Aberrant trophoblast differentiation, resulting in an abnormal number and proportion of the placental cell types, can severely impact the formation of this organ, resulting in placental insufficiency and pregnancy complications. Recent studies have highlighted the critical role of invasive cell lineage formation in uterine transformation, as insufficient cell differentiation has been associated with poor artery remodeling and the development of preeclampsia [14]. The trajectory of trophoblast differentiation was also found to be reconstructed in the preeclamptic placenta [15]. Consistently, specific cell markers of invasive trophoblast cells have been shown to be differentially expressed by the placenta under preeclamptic conditions, suggesting obstructed cell differentiation [16,17,18]. Moreover, inhibiting trophoblast differentiation has been shown to decrease spiral artery remodeling in vitro [19]. Collectively, existing data suggest that obstructed trophoblast differentiation is involved in the etiology of these pregnancy complications. Therefore, gaining a comprehensive understanding of the known developmental processes, mechanistic regulation, and identification of biomarkers for placental cell differentiation is crucial for evaluating the development and progression of placenta-induced diseases and could provide a basis for predicting these gestational disorders. This knowledge will not only shed light on the underlying mechanisms but also provide valuable insights for future research endeavors in this field.

## 2. Developmental Processes of the Placenta

The placenta plays an important role in supporting the growth and vitality of the developing offspring, and proper development of this organ is crucial for its functionality. In humans, the placenta consists of invasive and supportive trophoblast cells, and a specialized vascular network that arises through extensive villous branching and vasculogenic processes [20]. The human placenta is a hemochorial placenta with tree-like structures, known as chorionic villi, suspended in a free-flowing pool of maternal blood [21,22]. In mice, the placenta is organized into three distinct layers—the maternal decidua, the junctional zone, and the labyrinth [23,24]. The mouse placenta is also characterized as a hemochorial placenta and shares similar functionalities and developmental processes with the human placenta. 

### 2.1. Placental Cell Types in the Human and Mouse Placenta

#### 2.1.1. Distinct Types of Trophoblast Cells in the Human Placenta

**Figure 1:**
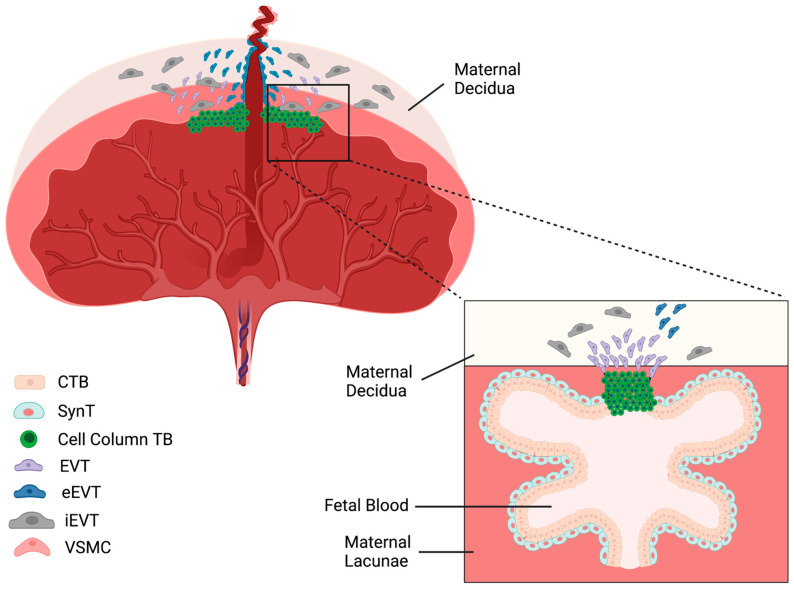
Structure of the human placenta. The decidua is the site of the maternal spiral artery attachment and is invaded by extravillous trophoblast cells for vessel remodeling and anchoring the placenta to the uterus. From the base of the placenta, chorionic villi emerge and expand to house the fetal capillaries. These tree-like villous structures suspend in a free-flowing pool of maternal blood, known as the lacunae. The maternal and fetal blood spaces are separated by two layers of cells, which house transporters and diffusive abilities, that facilitate the transport of nutrients and oxygen from the maternal to fetal circulation. Cytotrophoblasts (CTB); syncytiotrophoblasts (SynT); trophoblasts (TB); extravillous trophoblasts (EVT); endovascular extravillous trophoblasts (eEVT); interstitial extravillous trophoblasts (iEVT); vascular smooth muscle cells (VSMC).

The placenta in humans is composed of three distinct types of trophoblast cells: cytotrophoblast cells, syncytiotrophoblast cells, and extravillous trophoblast cells (Figure 1). Together, the cytotrophoblast and syncytiotrophoblast cells form the placental barrier that prevents maternal and fetal circulations from directly interacting [25]. The cytotrophoblast cells surround the fetal blood vessels within the placental villi and act as a stem cell pool to regenerate the syncytiotrophoblasts and extravillous trophoblasts [26,27]. Furthermore, these cells have a high metabolic activity, contributing significantly to the energy production necessary for placental maintenance [25]. Recent research has revealed three subtypes of cytotrophoblast cells in the first- and second-trimester placenta, with the first being a proliferative subtype that serves as a potential pool of replenishing cells [28]. The second and third subtypes are both non-proliferative, but the second subtype is the progenitor pool for the syncytiotrophoblast cells [28].

The syncytiotrophoblast cells form a monolayer along the epithelium of the villous structure, as they come in contact with the maternal blood [25]. These cells secrete various protein and steroid hormones, and are responsible for much of the nutrient transport between the fetal and maternal circulations [25].

On the other hand, the extravillous trophoblast cells are the invasive cells of the human placenta. They mainly arise from the cell column trophoblasts that detach from the anchoring placental villi [29]. These cells have a migratory function, as they invade the uterine wall to establish contact with the maternal circulation [30]. The interstitial extravillous trophoblast cells invade the uterine wall to anchor the placenta to the uterus, while the endovascular extravillous trophoblasts invade the maternal spiral arteries and replace the endothelial lining, remodeling the vessels and allowing for adequate blood transport into the placenta [31].

#### 2.1.2. Distinct Types of Trophoblast Cells in the Mouse Placenta

**Figure 2:**
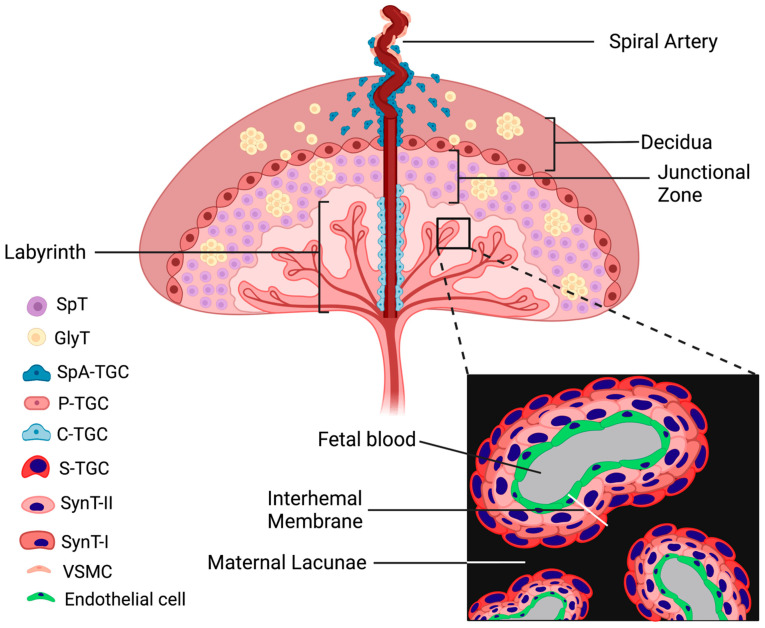
Structure of the mouse placenta. The mature placenta comprises 3 layers, the maternal decidua, the junctional zone, and the labyrinth. The decidua is the site of maternal spiral artery remodeling and uterine attachment by the invasive trophoblast cells. The junctional zone is the intermediate layer that supports the developing labyrinth through its endocrine role. The labyrinth houses the fetal capillaries and maternal lacunae, two distinct blood spaces that are separated by the interhemal membrane. Spongiotrophoblasts (SpT); glycogen trophoblasts (GlyT); spiral artery trophoblast giant cells (SpA-TGC); parietal trophoblast giant cells (P-TGC); canal trophoblast giant cells (C-TGC); sinusoidal trophoblast giant cells (S-TGC); syncytiotrophoblasts (SynT); vascular smooth muscle cells (VSMC).

Distinct types of trophoblast cells exist in both the mouse and human placenta, and they differ structurally (Figure 2). In the mouse placenta, spiral artery remodeling takes place in the decidua, allowing for the vasodilation of maternal vessels to supply adequate blood to the placenta [32]. This leads to the establishment of a central artery that shunts maternal blood to the base of the placenta and into the labyrinth layer, which houses the placental vascular network [20]. The junctional zone, or intermediate layer, has a structural role and is believed to provide nutrients and hormones to support the developing labyrinth [20].

In the mouse placenta, there are two lineages of trophoblast cells, the invasive and structural cells, which arise from the trophoblast stem progenitor cells [21]. Trophoblast giant cells are the invasive cells responsible for invading the maternal vasculature and transforming the spiral arteries into high-velocity, low-resistant vessels [32]. Four sub-types of trophoblast giant cells exist, including spiral artery trophoblast giant cells and parietal trophoblast giant cells, which reside in the decidual layer [33,34,35,36]. The canal trophoblast giant cells and sinusoidal trophoblast giant cells are in the labyrinth [32]. The structural cells of the placenta include those located within the junctional zone and the remaining cells of the labyrinth. The spongiotrophoblast and glycogen trophoblast cells are within the junctional zone. The spongiotrophoblast cells are thought to contribute a supportive role to the developing labyrinth and feed into the trophoblast giant cell progenitor pool [37,38], while glycogen cells, which arise from the spongiotrophoblast cells, are thought to accumulate glycogen and can migrate into the maternal decidua for spiral artery remodeling [39]. The two different types of syncytiotrophoblast cells, syncytiotrophoblast-I and syncytiotrophoblast-II, are located within the labyrinth zone [40]. These cells function in the material exchange between the mother and fetus and, along with the sinusoidal trophoblast giant cells, make up the interhemal membrane [40,41]. However, the specific functions of some of these cells are not well understood.

#### 2.1.3. Comparative Roles of Cell Types in the Mouse and Human Placentas

Although the mouse and human placentas have many different cell types, they perform similar functions. In the mouse placenta, the trophoblast stem progenitor cells are similar to the cytotrophoblast cells in the human placenta, as they both maintain stemness and give rise to other trophoblast cell types [36]. The spongiotrophoblasts in the mouse placenta are comparable to the cell column trophoblast cells in the human placenta, as they both differentiate into invasive trophoblast cells [36,38]. The trophoblast giant cells and glycogen cells in the mouse placenta are similar to the extravillous trophoblast cells in the human placenta. Specifically, the parietal trophoblast giant cells and glycogen trophoblast cells in the mouse placenta resemble the interstitial extravillous trophoblast cells in the human placenta, as they anchor the placenta to the uterine wall [36]. Furthermore, the spiral artery trophoblast giant cells in the mouse placenta are equivalent to the endovascular extravillous trophoblast cells in the human placenta, as they both function in maternal vascular invasion and spiral artery remodeling [36]. Finally, the syncytiotrophoblast-I and -II cells in the mouse placenta are similar to the syncytiotrophoblast cells in the human placenta, as they both act as a barrier between the maternal and fetal circulations and facilitate nutrient transport [42]. These similarities make the mouse placenta a valuable model for understanding the development and function of the human placenta and for investigating placental complications and diseases in pregnancy.

### 2.2. Differentiation of Trophoblast Cells

**Figure 3:**
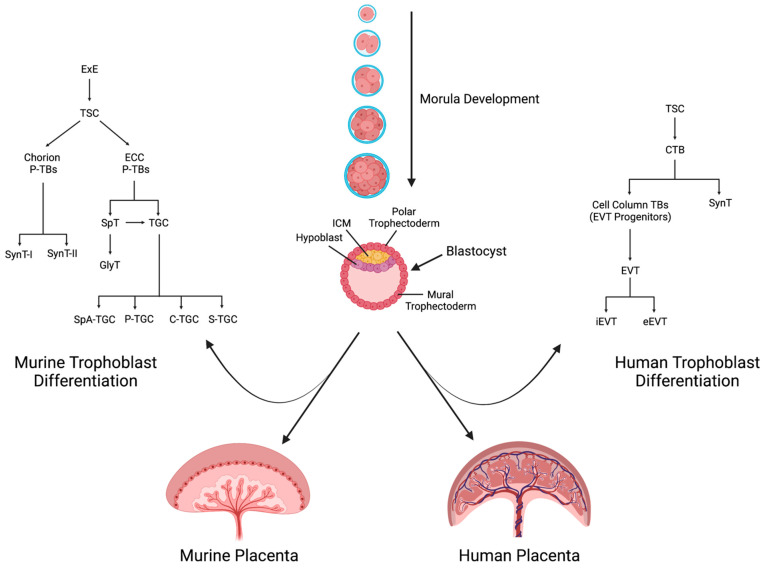
Lineage tracing of trophoblast differentiation. From the polar trophectoderm, trophoblast stem cells form and give rise to progenitor trophoblasts. These further differentiate into the structural, supportive, functional, and invasive cell types of the placenta. Extraembryonic ectoderm (ExE); trophoblast stem cells (TSC); ectoplacental cone (ECC); progenitor trophoblast cells (P-TBs).

Placental development begins early in gestation when the cells of the morula make their first developmental choice, either to become stem cells of the inner cell mass or the trophectoderm. The inner cell mass then develops the embryo, while the trophectoderm forms the placenta [40]. Importantly, the polar trophectoderm, which is the portion of the trophectoderm that comes into direct contact with the inner cell mass, gives rise to the majority of the mature placenta (Figure 3) [40].

#### 2.2.1. The Developmental Stages of Human Trophoblast Differentiation

In humans, early trophoblast stem cells are derived from the trophectoderm and express critical markers of trophoblast identity and self-renewal, including Krt8, Gata3, Tead4, and Tp63 [43,44,45]. These cells then differentiate into cytotrophoblasts or the multinuclear primitive syncytium, which become the first invasive placental cell types before the formation of the extravillous trophoblasts [27,46,47]. The proliferative cytotrophoblasts then form primary villi, and continuous proliferation and fusion of the developing villous cytotrophoblasts result in the formation of secondary villi [48]. The tertiary villi contain the fetal blood vessels and allow for materno-fetal exchange [48]. Concurrently, the proliferating cytotrophoblasts differentiate into invasive extravillous trophoblast cells [48]. As villous maturity is accomplished, the cytotrophoblasts within the tips of the anchoring villi form rows of proliferative cell column trophoblasts, serving as a progenitor pool for differentiating extravillous trophoblasts [48]. 

The extravillous trophoblasts further differentiate into interstitial extravillous trophoblasts, which invade the decidual stroma, and endovascular extravillous trophoblasts, which invade the maternal spiral arteries [49,50]. As extravillous trophoblasts invade, progenitor extravillous trophoblasts form in the proximal cell columns of anchoring villi in the placenta. These progenitors then stop proliferating within the distal cell columns and differentiate into interstitial extravillous trophoblasts or endovascular extravillous trophoblasts, which, respectively, invade the maternal decidual stroma and its vessels [51]. Shortly after implantation, the endovascular extravillous trophoblasts occlude the maternal spiral arteries, preventing premature oxygen delivery to the placental villi and reducing fetal oxidative stress [52]. Around the 10th week of pregnancy, the interstitial and endovascular extravillous trophoblasts collaborate to remodel the arteries and establish proper blood flow in the placenta [53]. Although the process of trophoblast differentiation in the human placenta is well-defined, the transcriptomic profile involved in the formation of the cell types is not fully understood, mainly due to the challenges in obtaining these organs from pregnancies.

#### 2.2.2. The Developmental Stages of Mouse Trophoblast Differentiation 

While the development of the human placenta has been difficult to examine, due to limited access to human organs during pregnancy, the mouse placenta has been extensively studied, and its differentiation processes are well understood.

The initiation of murine placentation involves paracrine growth signals from the inner cell mass, specifically Fgf4, which induces stemness and the proliferation of trophoblast stem cells through Fgfr2 signaling of Cdx2 and Eomes transcription factors [54,55]. During development, some of these cells maintain Cdx2 and Eomes expression, giving rise to the chorion and eventually developing into the labyrinth layer [40]. As trophoblast cells move away from the developing embryo, Fgf4 signaling decreases, and differentiation follows. The ectoplacental cone then develops, allowing for the transcription of Mash2, which provokes the development of trophoblast progenitor cells from the stem cell pool [56]. This step is crucial for placental development, and deletion of Mash2 results in a significant reduction in ectoplacental cone cells and a loss of spongiotrophoblasts [56]. 

The ectoplacental cone also begins to express Tpbpα, a key marker gene for the junctional zone and invasive trophoblast giant cells [57]. Ablation of Tpbpα results in trophoblast invasion deficiencies and defective spiral artery remodeling [58]. From the ectoplacental cone, the cells further differentiate into either spongiotrophoblasts or trophoblast giant cells. Spongiotrophoblast cells retain Tpbpα expression and can feed into the trophoblast giant cell pool or further differentiate into glycogen trophoblast cells [40]. Trophoblast giant cells begin to differentiate upon the suppression of Mash2 and the expression of Hand1, which antagonizes Mash2 function and promotes trophoblast giant cell formation [40,59]. Mash2, which is a basic helix–loop–helix (bHLH) transcription factor, interacts with E proteins, Itf2 and Alf1, to bind to DNA and initiate transcription and maintain the giant cell precursors [60]. Mutations in Hand1 induce defects in trophoblast giant cell differentiation and labyrinth vascularization, resulting in significant growth restriction and fetal demise in some cases [61,62]. Interestingly, trophoblast giant cells have been shown to have differing precursors, sourced from outside the ectoplacental cone and spongiotrophoblast cells. In a study that crossed female Tpbpα-Cre mice with male Z/AP dual reporter mice, the findings were able to show that all the spiral artery trophoblast giant cells originated from Tpbpα+ precursors, while about 50% of the parietal and canal trophoblast giant cells, and 100% of the sinusoidal trophoblast giant cells, arose from Tpbpα− origins [40]. This finding underscores the importance of mechanistic regulation and proper signaling stimuli to drive trophoblast differentiation.

## 3. Important Cell Signaling in Trophoblast Differentiation 

### 3.1. FGF4 Signaling Pathway 

Fibroblast growth factors (Fgfs) are a family of proteins that are functionally relevant in many different biological processes, including embryonic development and homeostasis [63]. Fgfs act through four subtypes of receptors, Fgfr1–4, which are transmembrane tyrosine kinases, and induce their responses through downstream signaling pathways, such as RAS/MAPK, PI3K/AKT, and PLCγ [64,65]. During embryonic development, the Fgf signaling pathway is important for cell survival, proliferation, differentiation, and migration [63]. Specifically, Fgfs are prominent regulators of pluripotency of hematopoietic and mesenchymal stem cells [63]. 

#### 3.1.1. FGF4 

During the early stages of placental development, the extraembryonic ectoderm is formed, and it consists of self-renewing trophoblast stem cells that are essential to provide the ectoplacental cone with progenitor cells for trophoblast giant cells and spongiotrophoblasts [66,67,68]. Fgf4 signals from the inner cell mass support the retention of the capacity of these trophoblast stem cells to proliferate and self-renew [69]. Trophoblast differentiation is important for the establishment of the placenta, but initially, obtaining adequate stemness is necessary. The suppression of ErrB, Eomes, and Cdx2, which are transcription factors expressed by trophoblast stem cells, and an increase in Mash2, indicating differentiation of these stem cells to trophoblast progenitor cells, occur as Fgf4 signaling is lost [69]. Interestingly, an in vitro study showed that Fgf4 signaling alone was not sufficient to increase ErrB, Eomes, or Cdx2 in the trophoblast cells. However, the addition of Nodal with Fgf4 significantly enhanced the expression of these transcription factors, suggesting that Nodal is an important agonist within Fgf4 signaling activity to maintain trophoblast proliferation before differentiation [69].

#### 3.1.2. Cross-Talk between FGF4 and Other Signaling Pathways 

Fgf4 signaling has notable interactions with other signaling pathways to induce a specific response. As previously mentioned, the RAS/MAPK pathway is a downstream target of Fgf. The maintenance of the trophoblast stem cell compartment by Ras/Mapk/Erk is thought to be Fgf-dependent [70]. In trophoblast stem cells, Fgf4 was found to activate RAS/ERK through Shp2, which is a nonreceptor protein-tyrosine phosphatase important for the normal activation of this pathway [71]. This then destabilizes the apoptotic factor, Bim, further allowing for trophoblast stem cell proliferation and survival [72]. Furthermore, trophoblast stem cells treated by Fgf4 displayed increased differentiation and evidence of EMT upon the inactivation of mitogen-activated protein kinase kinase (MEKK4), as well as a decreased activation of Jnk and p38. This indicates that Fgf4 works with the RAS/MAPK signaling pathway for the maintenance and self-renewal of trophoblast stem cells in an undifferentiated state [73]. 

Fgf4 has other known interactions in terms of function in early trophoblast development. In trophectoderm cells, which are the precursors for the developing placenta, there was a significant activation of p-AKT upon Fgf4 incubation, increasing cell migration, which is important for placental implantation [74]. Additionally, in trophoblast stem cells, the addition of Activin to Fgf4-treated cells increasingly promotes cell proliferation and self-renewal [75]. This response was similar in other experiments, in which the cells were treated with Fgf4+Nodal or Fgf4+TGFβ [69,75]. Together, this data indicates that Fgf4 works synergistically with other signaling pathways to promote cell proliferation, survival, and migration during the early stages of placental development. 

### 3.2. IGF-1 Pathway

IGF-1, or insulin-like growth factor 1, is a peptide hormone that belongs to a family of similar hormones, including IGF-2. Its production is triggered by the release of growth hormone (GH) in the body [76]. Apart from its role in promoting growth and development, IGF-1 plays a crucial part in regulating various metabolic processes, such as protein synthesis and breakdown, glucose uptake, and cellular metabolism. The availability and distribution of IGF-1 in the bloodstream is controlled by a group of proteins called IGFBPs, or IGF-binding proteins. These proteins interact with IGF-1 and can either enhance or inhibit its activity, depending on their specific interactions. Furthermore, IGF-1 itself can exert feedback regulation on its own production, meaning that high levels of IGF-1 can suppress the release of GH, thereby reducing further IGF-1 production [77].

#### 3.2.1. IGF-1

IGF-1 has been identified as an essential factor for fetal survival. Studies on IGF-1 null mice have shown that approximately 95% of embryos lacking IGF-1 do not survive, while the few surviving pups exhibit severe growth retardation and developmental defects affecting various organs, including the brain, muscle, bone, and lungs [78]. In the context of fetal growth and development, IGF-1 is locally produced in the placenta and acts as a critical regulator. It stimulates the production and release of placental hormones, like hCG and progesterone. Additionally, IGF-1 plays a role in promoting the proliferation and differentiation of trophoblast cells, supporting proper placental formation by facilitating angiogenesis, the process of forming new blood vessels within the placenta. The placenta itself expresses IGF-1 receptors and IGF-1 binding proteins (IGFBPs), which are involved in regulating the distribution and activity of IGF-1 [79].

#### 3.2.2. Cross-Talk between IGF-1 and Other Signaling Pathways 

IGF-1 has been the subject of extensive research due to its involvement in various crucial signaling pathways, contributing to development and proliferation. One prominent pathway activated by IGF-1 is the PI3K/AKT pathway. When IGF-1 binds to IGF-1R, it triggers the recruitment and activation of PI3K, subsequently leading to the activation of AKT. This pathway is known to regulate the differentiation of different trophoblast cell types, including invasive giant cells and cytotrophoblasts [25,80]. Additionally, in addition to directly impacting trophoblast differentiation, the IGF-1/AKT/PI3K axis also plays a role in the secretion of β-hCG and progesterone, which are involved in regulating the syncytialization of cytotrophoblasts [81,82] The multifaceted function of IGF-1 in interacting with these essential signaling pathways underscores its significance in regulating trophoblast development and the associated hormonal activities. 

IGF signaling has been identified as a significant influencer of the Wnt pathway in trophoblast cells. IGFs have been demonstrated to stimulate Wnt signaling through various mechanisms [83]. Specifically, IGF-1 can enhance the expression of Wnt ligands, stabilize β-catenin, and promote the nuclear translocation of β-catenin, a key effector of canonical Wnt signaling [84,85]. The canonical Wnt pathway, mediated by β-catenin, plays a crucial role in the formation and maintenance of the trophoblast giant cell layer and syncytiotrophoblasts [86].

While IGF-1 interacts with several essential pathways involved in trophoblast differentiation, its primary function lies in promoting the formation of syncytiotrophoblasts from cytotrophoblasts and facilitating the differentiation of invasive extravillous trophoblasts [82]. Consequently, IGF-1 presents an intriguing target for future investigations into pregnancy complications associated with immature villus development and improper implantation. Understanding the role of IGF-1 in these processes could offer valuable insight for the development of potential therapeutic interventions. 

### 3.3. TGF-β Signaling

The TGF-β family comprises numerous members, including TGF-β itself, bone morphogenetic proteins (BMPs), activins, inhibins, and Nodal [87]. These members activate the Smad family via activin receptor-like kinases upon binding to their receptors [87]. Upon phosphorylation, the Smad proteins generally form dimers with the mediator protein, Smad4, and translocate to the nucleus to activate transcription [87]. Throughout placental development, the TGF-β family regulates trophoblast proliferation, differentiation, migration, and invasion [88]. Importantly, dysregulated TGF-β signaling has been implicated in preeclampsia, a pregnancy complication resulting from inadequate spiral artery remodeling and placental dysfunction [89]. Consequently, this pathway has been extensively studied and is known to have a crucial role in proper placentation.

#### 3.3.1. TGF-β

TGF-β plays a crucial role in trophoblast proliferation, migration, and invasion. It consists of three isoforms, namely TGF-β1, TGF-β2, and TGF-β3 [88]. When activated, these isoforms cause the phosphorylation of Smad 2 or 3, forming a complex with Smad 4 that translocates to the nucleus and regulates gene transcription [90]. Exposing rat trophoblast stem cells to TGF-β resulted in increased expression of Tpbpα, Prl3d1, Prl3b1, and Prl2c2, indicating increased differentiation of spongiotrophoblasts and trophoblast giant cells. Conversely, adding Activin to these cells led to a labyrinth cell fate [75].

However, in humans, TGF-β has a different effect: it suppresses the formation of extravillous trophoblast and promotes the syncytialization of cytotrophoblast cells [91]. Treatment of SM10 cells with TGF-β resulted in a significant increase in p-Smad2 levels, leading to labyrinth cell formation [92]. Additionally, when villous explants from first-trimester human placentas were exposed to TGF-β, the outgrowth effect induced by Activin was reversed [93]. On the other hand, the expression of TGIF-1, a suppressor of TGF-β, is significantly increased in differentiated syncytiotrophoblasts from cytotrophoblasts in both first- and third-trimester human placentas. Decreased TGIF-1 expression is associated with decreased differentiation of syncytiotrophoblasts and a smaller syncytium [94]. 

TGF-β plays a crucial role in the differentiation and maturation of various extravillous trophoblast subtypes. Phosphorylated Smad2 was found to be mainly present in the HLA-G negative proximal cell column of cytotrophoblasts, while phosphorylated Smad3 signals were stronger in the extravillous trophoblast region of the human placenta [90]. In TGF-β inhibitory conditions, there was a significant increase in the concentration of progenitor extravillous trophoblasts, as indicated by the upregulation of HLA-G and VE-cadherin. Silencing of Smad2 in HTR-8/SVneo cells resulted in significant outgrowth, increased expression of MMP1 and VE-cadherin, accelerated migration, and increased invasion, whereas silencing of Smad3 had the opposite effect, indicating that Smad2 inhibits while Smad3 stimulates extravillous trophoblast differentiation and invasion [90]. However, with the provision of TGF-β, a significant increase in the expression of DAO, PAPPA2, and FN1, and a decrease in VE-cadherin were observed, indicating the differentiation of progenitor extravillous trophoblasts into interstitial extravillous trophoblasts [51]. TGF-β was also found to induce the differentiation of progenitor extravillous trophoblasts into interstitial extravillous trophoblasts through Smad3 activation [51]. 

#### 3.3.2. Nodal

During placental development, Nodal plays a crucial role in regulating invasive trophoblast differentiation. In mouse mutants, lack of Nodal resulted in abnormal placentation with an excessive number of trophoblast giant cells [95]. Moreover, the addition of Nodal inhibited trophoblast giant cell formation and decreased PL-I and PL-II gene promoter activity in Rcho-1 cells and trophoblast stem cells [95]. Conversely, the inhibition of Nodal signaling in human embryonic stem-cell-derived trophoblasts led to EMT and invasive cytotrophoblast formation while promoting syncytiotrophoblast formation [96]. In the placenta, Stox1 is a transcription factor that regulates genes, increasing trophoblast proliferation and decreasing invasion, and is regulated by IGF-1 [97]. Interestingly, Nodal addition to first-trimester extravillous trophoblasts led to increased IGF-1 secretion, resulting in increased expression of Stox1 [97]. Overall, the current literature suggests that Nodal is crucial for maintaining the trophoblast stem cell pool and promoting labyrinth formation during development.

#### 3.3.3. Activin

The role of Activin signaling is also crucial in the development of the placenta, especially in trophoblast differentiation. Similar to Nodal, the absence of Activin signaling promotes the formation of invasive trophoblasts in vitro [96]. Conversely, the treatment of trophoblast stem cells with exogenous Activin results in a significant increase in Gcm1 and Synb expression, indicative of syncytiotrophoblast differentiation, as well as a significant decrease in or delayed expression of Tpbpα, Prl3d1, Prl3b1, and Prl2c2, which are markers for spongiotrophoblasts and trophoblast giant cells, when Fgf4 is removed from the medium [75]. In contrast, the provision of Activin to villous explants from first-trimester human placentas leads to substantial outgrowth of cells from the villous tip, accompanied by increased expression of HLA-G, MMP-9, and fibronectin by the cells of the villous outgrowth [93], indicative of cytotrophoblast differentiation into invasive extravillous trophoblasts. These findings are consistent with other results showing that Activin promotes extravillous trophoblast differentiation and invasion [93,98]. However, the addition of Activin to SM-10 cells does not effectively promote differentiation [92]. Although the data on the role of Activin in trophoblast differentiation are conflicting, proper regulation of this signaling pathway is essential for placental development.

### 3.4. Hypoxia and HIF-1

In the early stages of gestation, the trophoblast cells are exposed to a low-oxygen environment due to limited maternal blood flow to the placenta, which is crucial for protecting the developing embryo against free radicals [99]. However, as the embryo grows, sufficient oxygen is required for normal development, and this must be provided by the maternal circulation. Hence, low oxygen levels are a key driver of placentation. Under hypoxic conditions, the expression of hypoxia-inducible factor-1 (HIF-1) increases. HIF-1 is a heterodimeric protein composed of alpha and beta subunits [100]. These subunits become stabilized and then move into the nucleus, where they bind with the HIF-1 beta subunit, ARNT, to form a transcriptionally active complex that induces the expression of genes associated with glycolysis, red blood cell production, and angiogenesis [99,100]. Once normoxia is restored, the HIF-1 subunits are degraded [101].

Research has demonstrated that oxygen tension can impact the differentiation of trophoblast cells [99]. In mice with a mutation in HIF-1α, labyrinth vascularization, trophoblast invasion, and Tpbp expression were significantly impaired [101]. Hypoxic conditions (3% O_2_) also led to an increase in Tpbp mRNA levels and a reduction in Plf and Hand1 expression in TS cells, indicating that low oxygen favors spongiotrophoblasts over trophoblast giant cell differentiation [101]. Low-oxygen tension also increased the proportion of HLA-G+ extravillous trophoblasts in cultured cytotrophoblasts from first-trimester human placentas, accompanied by changes in MMP2 and hCG secretion, which are markers of extravillous and syncytiotrophoblast formation and functionality, respectively [100]. However, the loss of ARNT in these cytotrophoblasts abrogated these effects [100]. Human villous explants from first-trimester placentas cultured under hypoxic conditions exhibited increased extravillous trophoblast outgrowth from the distal end of the villous tip, which was accompanied by increased MMP2 activity and the expression of HIF-1α and TGF-β3 transcripts [102]. Inhibition of HIF-1α in the hypoxic explants downregulated TGF-β3, indicating that TGF-β signaling may regulate the role of hypoxia in trophoblast differentiation [102].

Spiral artery remodeling regulates oxygen tension in the placenta. Prior to trophoblast differentiation, immune cells are recruited to the placentation site to initiate this process. Uterine natural killer cells play a significant role in this process, promoting slight vessel remodeling by decreasing arterial smooth muscle wall thickness and increasing vessel lumen diameter [103]. These natural killer cells also have a profound impact on the development and function of the invasive trophoblast cell lineage, as they secrete cytokines, have direct cell–cell interactions, and control oxygen tension to initiate differentiation [103]. The absence of natural killer cells at the maternal–fetal interface results in transient hypoxia, leading to poor placentation [103]. Therefore, natural killer cells are important in regulating oxygen concentration and inducing the expression of HIF-1, leading to the differentiation of invasive trophoblast cells and inhibiting the formation of syncytiotrophoblasts [103]. Together, these findings suggest that hypoxia plays an important role in trophoblast differentiation, particularly of the invasive cell lineage, and that spiral artery remodeling and natural killer cells are key regulators of oxygen tension and trophoblast differentiation.

### 3.5. Retinoic Acid Signaling Pathway

Retinoic acid (RA) is the bioactive form of vitamin A, which exerts its effects by binding to two different receptors, the retinoic acid receptors (RARs) and the retinoid X receptors (RXRs) [104]. These receptors function as transcription factors and modulate gene expression by binding to specific DNA response elements in their target genes [105]. RARs and RXRs are expressed in the placenta, particularly within syncytiotrophoblasts, and an RXR deficiency leads to abnormal labyrinth development [105,106]. In vitro studies have demonstrated that RA increases the production of hCG and hPL, which occurs during the differentiation of cytotrophoblasts to syncytiotrophoblasts [107]. Moreover, RXR levels increase during trophoblast differentiation, indicating RA’s involvement in this cellular process [107].

When trophoblast stem cells are cultured in the presence of FGF4, they remain undifferentiated. However, upon the addition of retinoic acid (RA) to the media, the cells show reduced growth and changes in morphology within 48 h [104]. This is accompanied by a significant decrease in Id-2, which is a negative regulator of bHLH transcription factors and is highly expressed in trophoblast progenitors [104,108]. As the cells differentiate upon RA treatment, there is a significant increase in Pl-1, a marker for trophoblast giant cells, while Tpbpα becomes undetectable [104]. Additionally, the proportion of Tpbp-positive cells is significantly reduced upon RA treatment [104]. In vivo studies have also shown that injection of RA into pregnant mice at E6.5 and 7.5 causes the placental giant cell layer to thicken and the number of spongiotrophoblasts to decrease [104]. Similarly, trophoblast cells isolated from the rat placental junctional zone on day 13 of pregnancy exhibit decreased expression of PLP-C and Pl-IV, proteins expressed by spongiotrophoblasts, upon RA treatment [109]. These findings suggest that RA promotes trophoblast giant cell differentiation and inhibits spongiotrophoblast formation.

In contrast, the expression of RA receptors, including RXR, is prominent in differentiating syncytiotrophoblasts [105]. As mentioned earlier, the RXR mutant exhibits defective labyrinth development, indicating the significance of RA signaling in the formation of these cells. When human placental cytotrophoblasts were treated with the RXR agonist, BMS649, or the PPAR-γ ligand, rosiglitazone, there was a considerable increase in hCG and hPL secretion, indicative of syncytiotrophoblast differentiation [105]. PPAR-γ forms heterodimers with RXRs, which bind to PPAR-responsive elements within the promoters of PPAR-γ target genes [110]. Syncytiotrophoblast cells also show high expression of PPAR-γ, which is similarly expressed to RXR in their nuclei [105]. Furthermore, trophoblast cells treated with PPAR-γ induced syncytiotrophoblast cell formation [111]. Interestingly, exposure of cultured extravillous trophoblasts to PPAR-γ or RXR antagonists leads to increased trophoblast invasion, indicating that the activation of receptors mediates the impact of RA signaling on trophoblast differentiation [112]. Although the response of RA signaling on trophoblast formation varies, it still plays a vital role in placenta development.

## 4. Epigenetic Regulation of Trophoblast Differentiation 

Trophoblast differentiation is influenced by epigenetic modifications [113]. Trophoblast cells are globally hypomethylated compared to the inner cell mass, and DNA methylation of the transcription factor Elf5 controls the lineage fate of cells within the early blastocyst. Elf5 is hypomethylated in the trophoblast compartment and positively reinforces trophoblast fate and trophoblast stem cell proliferation through a feedback loop with Cdx2 and Eomes [114]. Embryo-specific genes Oct4 and Nanog are globally methylated and repressed in trophoblast stem cells, while Plet1 is hypomethylated and promotes cell lineage specification [115,116]. Overexpression of Plet1 in trophoblast cells accelerates differentiation towards ectoplacental cone-like trophoblasts, specifically trophoblast giant cells, while downregulating syncytiotrophoblast cell markers [117].

During normal placental development, downregulation of Dnmt1, Dnmt3a, and Dnmt3b in cytotrophoblast cells leads to hypomethylation of syncytin-1, resulting in the differentiation of syncytiotrophoblasts [118]. Overexpression of Dnmt3a in cytotrophoblast cells induces the downregulation of syncytin-1, leading to defective syncytiotrophoblast formation and placental abnormalities [119]. The differentiation of trophoblast stem cells to the invasive cell lineage is also epigenetically regulated [120]. Hypermethylation of E-cadherin, Krt7, Dnmt1, and Wnt signaling inhibitors, as well as hypomethylation of Snail and Slug, regulate the development and functionality of invasive trophoblasts, as many of these signaling members are involved in trophoblast invasion [121,122,123,124,125,126]. Treatment of invasive trophoblast cells with HDAC inhibitors inhibits invasion by causing chromatin to decondense [127,128,129].

Trophoblast differentiation is also modulated by microRNAs. Overexpression of miR-218-1, which is a precursor for miR-218-5p, in vitro, led to significant increases in markers of endovascular extravillous trophoblast differentiation and invasion, including Mmp1, Pecam1, VE-cadherin, IL-8, and IL-1β [19]. IL-1β has been found to promote endovascular extravillous trophoblast differentiation, and interestingly, TGF-β signaling, through the activation of Smad2, reduces the levels of IL-1β protein and extravillous trophoblast formation [130]. The microRNA, miR-218-5p, targets and inhibits TGF-β signaling, which increases IL-1β expression and cell differentiation during endovascular extravillous trophoblast differentiation [19,130]. Additionally, miR-378a-5p downregulates Ccng2 expression and inhibits syncytiotrophoblast differentiation [131]. These findings demonstrate the crucial role of epigenetic regulation in the differentiation of trophoblast cells.

## 5. Biomarkers of Trophoblast Cells 

Single-cell RNA-seq technology has become a popular method for analyzing RNA transcripts and gene expression in individual cells, revealing the composition of different cell types within biological tissues [132]. This approach allows for the molecular characterization of individual cells, the identification of various cell populations present in the organ, and an understanding of cellular functions and interactions that contribute to tissue function in vivo [132]. In the context of investigating the placenta and its spatiotemporal gene expression patterns throughout development, single-cell RNA-seq can be an invaluable tool. However, for this technology to be effective, it is crucial to accurately define cell populations based on known biomarkers of trophoblast cells (see Table 1 and Table 2).

Cytokeratin 8 and 18 (Krt8, Krt18) are expressed in all trophoblast cells within the human placenta [96]. Recent studies have shown that Spint1 and Hai-1 are expressed in cytotrophoblast cells, which are negative for HLA-G [51,90]. Upon differentiation into syncytiotrophoblasts, these cells increase their production of hCG and hPL and express Syncytin-1 and Cyp19a1 [96,105,113,120]. Extravillous trophoblast cells, which arise from cytotrophoblasts, undergo epithelial-to-mesenchymal transition and express VE-cadherin, as well as HLA-G, Notch1, Erbb2, Tcf-4, and Tead2 [51,96,100]. Interestingly, HLA-G polymorphisms have been associated with pre-eclamptic pregnancies, potentially due to the poor development of these invasive cell types [133,134]. Interstitial and endovascular extravillous trophoblast cells express specific markers, such as Pappa2, Dao, and Serpine1, and Itga1, Itga5, VE-Cadherin, and Pecam1, respectively, to distinguish them [51,130]. While markers have been identified for specific cell types, there is limited knowledge regarding their temporal and mechanistic regulation throughout placental development. Single-cell RNA-seq technology can help in identifying the spatiotemporal patterning of gene expression and the composition of different cell types within the placenta. However, the proper definition of cell populations based on known biomarkers of trophoblast cells is necessary for this technology to be effective.

As mentioned earlier, the mouse and human placentas exhibit many similarities in their organ function. However, structural differences exist between the two, including differences in the cell populations present in the tissues. Interestingly, the key markers of murine trophoblast cells are weakly detectable or absent in human trophoblast cells [43]. Despite these discrepancies, the cell populations in the mouse placenta are comparable to those in the human placenta. Therefore, using a murine model can be a valuable tool for studying placental development throughout pregnancy. This information can then be used to make inferences about the development of placenta-induced diseases, such as preeclampsia.

### 5.1. Extraembryonic Ectoderm, Ectoplacental Cone, and Chorion

During murine placental development, the extraembryonic ectoderm is one of the earliest structures to form and house trophoblast stem cells. These stem cells differentiate into cells of the ectoplacental cone or chorion, which form the junctional zone and labyrinth, respectively. Trophoblast stem cells express several transcription factors, including Cdx2, Eomes, Elf5, Essrb, and Id2 [55,135,136]. The chorion arises through the continued expression of Cdx2, Eomes, Essrb, Esx1, and Gcm1, with Gcm1 being a defining marker of chorionic trophoblast stem cells and labyrinth trophoblasts, starting at E7.5 [137]. In contrast, the differentiation of progenitor trophoblast cells in the ectoplacental cone is marked by decreased expression of trophoblast stem cell markers and increased expression of Mash2 (Ascl2) from E6.5 [138]. Additionally, cells of the ectoplacental cone express Dlx3 and Nr6a1, specifically at E7.5 [139], and secretin expression is localized in the ectoplacental cone beginning at E6.5 [138]. Furthermore, many progenitor cells of the ectoplacental cone express Tpbpα, which distinguishes them from those of the labyrinth layer [40]. Although there are structural differences between the mouse and human placenta, the use of a murine model can be beneficial in studying placental development and diseases, such as preeclampsia, due to similarities in cell populations.

### 5.2. Spongiotrophoblast Cells

The spongiotrophoblast cells differentiate from the ectoplacental cone by losing Mash2 expression but retaining Tpbpα expression. Unlike the trophoblast giant cells that differentiate from the ectoplacental cone, they do not express Hand1 [40]. These cells express cytokeratin 8 and 18 (Krt8, Krt18), which are proteins expressed by almost all types of trophoblast cells [139,140]. Trophoblast cells produce hormones called placental lactogens (Pls) or prolactins (Prls). Prl5a1 is produced specifically by spongiotrophoblast cells from E8.5 to 9.5, while Prl3c1, Prl3a1, Prl8a8, and Prl8a6 are produced specifically by spongiotrophoblast cells starting at E14.5 [141,142]. During pregnancy, spongiotrophoblasts also produce several other Prl genes, including Prl2b1, Prl2c5, Prl3b1, Prl7a1, Prl7a2, Prl7d1, and Prl8a9 [142], although these genes are not specific to spongiotrophoblasts.

### 5.3. Glycogen Trophoblast Cells 

Glycogen trophoblast cells emerge from spongiotrophoblast cells around E12.5 and are predominantly located in the junctional zone. These cells play a significant role in the production of IGF-2 during the second half of pregnancy. Tpbpα expression is maintained in these cells, along with the expression of Cdkn1c [143]. Protocadherin12 (Pcdh12) is a gene that is exclusively expressed in glycogen trophoblast cells [39]. Prl6a1, Prl2a1, Prl8a9, Prl7b1, Prl7c1, and Prl7d1 are also expressed by these cells, although they are not specific to them [142].

### 5.4. Trophoblast Giant Cells

The trophoblast giant cells arise from the ectoplacental cone upon the downregulation of Mash2 and upregulation of Hand1 and Stra13 [40,144]. Additionally, spongiotrophoblasts can contribute to the trophoblast giant cell population. Four distinct subtypes of trophoblast giant cells emerge after E9.5, including the parietal, spiral artery, canal, and sinusoidal trophoblast giant cells, each with unique functions in placental development and distinct locations within the organ [20].

The parietal trophoblast giant cells play a critical role in implantation and invade the decidua. These cells express Hand1, a common marker for all trophoblast giant cell subtypes. Interestingly, only about half of these cells express Tpbpα [38]. Prl3d1, also known as Pl-I, is specifically produced by parietal trophoblast giant cells, while Prl2b1, Prl7a1, and Prl3b1, also known as Pl-II, are produced by all trophoblast giant cell subtypes [142]. Prl4a1 is another prolactin gene predominantly expressed by parietal trophoblast giant cells, particularly before E12.5, and has been shown to modulate uterine natural killer cells, which play an essential role in initiating maternal artery remodeling and decidualization [145].

The spiral artery trophoblast giant cells, arising from Tpbpα+ precursors, secrete proteinases and vasodilators to regulate maternal spiral artery remodeling and blood flow into the placenta. Similar to glycogen trophoblasts, Prl6a1 and Prl7b1 are expressed by these giant cells [142]. Hand1 expression is the differentiating factor between these two cell types. Additionally, spiral artery trophoblast giant cells express Prl7d1 and Plf (Prl2c2), an angiogenic factor that stimulates cell migration [142]. 

The canal trophoblast giant cells are responsible for maternal vascular remodeling within the labyrinth and line the central artery that shunts maternal blood to the base of the labyrinth. About half of these cells arise from Tpbpα+ precursors, with Prl7b1 as the most specific marker. These cells also express Prl3b1, which is absent from spiral artery trophoblast giant cells. Like the other subtypes, canal trophoblast giant cells express Prl2c2 and Prl7d1 [142]. 

The trophoblast giant cells of the sinusoidal subtype have a unique function in the placenta: to regulate the activity of hormones and growth factors and facilitate the transfer of nutrients and oxygen between the mother and the fetus. These cells are located in the interhemal membrane of the labyrinth and do not originate from Tpbpα precursors, suggesting that they may have more similarities with chorion cells. The sinusoidal trophoblast giant cells express Prl2b1, Prl3b1, and Prl7d1, but the most distinctive marker of this cell type is Cathepsin Q (Ctsq), which is expressed from E11.5 to the end of gestation [38,142,146].

### 5.5. Syncytiotrophoblast Cells

The syncytiotrophoblast cells originate from the chorion during placental development in mice. There are two layers of these cells in the mouse placenta, known as syncytiotrophoblast-I and -II cells. These cells encounter fetal endothelial cells and play a crucial role in nutrient transport. Although tightly adhered to each other through tight junctions, these two cell layers differ in composition [137]. As syncytiotrophoblasts differentiate, Gcm1 expression is observed in cells closer to the fetal capillaries rather than those near the maternal lacunae, indicating that syncytiotrophoblast-II cells retain this expression [137]. Additionally, these cells express Cebpa and Synb starting from E8.5 [138]. In contrast, Syna expression is limited to syncytiotrophoblast-I cells and can be detected at E8.5 [138]. Notably, mice with a mutation in Syna exhibit impaired formation of the syncytiotrophoblast-I layer but retain the syncytiotrophoblast-II layer, highlighting its specific role in the development of this cell type [147].

## 6. Conclusions

In recent years, significant progress has been made in understanding the development and function of the placenta, particularly regarding trophoblast differentiation, which plays a critical role in placentation. Tight regulation of these cellular processes is essential, as aberrations can lead to poor maternal–fetal interface establishment, resulting in pregnancy complications, such as preeclampsia and placental insufficiency. While much progress has been made, knowledge gaps still exist. However, murine models offer valuable opportunities for experimentation, as their placental function closely resembles that of humans. Utilizing these models can provide insights into human placental development, especially during the later stages of pregnancy when complications tend to arise. Furthermore, leveraging technologies like single-cell RNA sequencing can yield valuable information on the molecular aspect of specific cell types and provide a better understanding of the tissue’s composition. These efforts can have a significant impact on improving pregnancy outcomes and maternal–fetal health.

## Figures and Tables

**Table 1:** Markers for trophoblasts in human placenta.

Cell Types	Specifics for scRNAseq ID		Additional Markers
	Positive	Negative	
Cytotrophoblasts	Krt8/18 Spint1 Hai-1	HLA-G	
Syncytiotrophoblasts	Krt8/18 hCG hPL Syncytin-1	HLA-G	Cyp19a1
Extravillous Trophoblasts	Krt8/18 HLA-G VE-Cadherin		Notch2, Erbb2, Tcf-4, Tead2
Interstitial Extravillous Trophoblasts	Krt8/18 Pappa2 Dao Serpine1		
Endovascular Extravillous Trophoblasts	Krt8/18 Itga1/5 VE-Cadherin Pecam1		

**Table 2:** Markers for trophoblasts in murine placenta.

Cell Types	Specifics for scRNAseq ID		Additional Markers
	Positive	Negative	
Spongiotrophoblasts	Krt8/18 Tpbpα	Hand1 Cdkn1c Pcdh12	Prl5a1 (E8.5-9.5); Prl3c1, 3a1, 8a8, 8a6 (E14.5-term)
Glycogen Trophoblasts	Krt8/18 Tpbpα Cdkn1c Pcdh12	Hand1	Prl6a1, 7b1, 7c1 (after E12.5)
Trophoblast Giant Cells	Krt8/18 Hand1 Tpbpα (+/−)		
Parietal Trophoblast Giant Cells	Krt8/18 Hand1 Tpbpα (+/−) Prl3d1	Prl6a1 Prl7b1 Prl7c1	Prl4a1 (before E12.5); Prl2b1, 7a1, 3b1
Spiral Artery Trophoblast Giant Cells	Krt8/18 Hand1 Tpbpα Prl6a1 Prl7b1	Prl3d1 Prl3b1	Prl7d1, 2c2
Canal Trophoblast Giant Cells	Krt8/18 Hand1 Tpbpα (+/−) Prl7b1 Prl3b1	Prl6a1	Prl2c2, 7d1
Sinusoidal Trophoblast Giant Cells	Krt8/18 Hand1 Ctsq	Tpbpα	Prl2b1, 3b1, 7d1
Syncytiotrophoblast-I	Krt8/18 Syna	Hand1 Tpbpα Gcm1 Synb Cebpa	
Syncytiotrophoblast-II	Krt8/18 Gcm1 Synb Cebpa	Hand1 Tpbpα Syna	

## Data Availability

No new data were generated.
